# Potential molecular mechanisms of chronic fatigue in long haul COVID and other viral diseases

**DOI:** 10.1186/s13027-023-00485-z

**Published:** 2023-02-07

**Authors:** Carl Gunnar Gottschalk, Daniel Peterson, Jan Armstrong, Konstance Knox, Avik Roy

**Affiliations:** 1 Simmaron Research INC, 948 Incline Way, Incline Village, NV 89451 USA; 2 Research and Development Laboratory, Department of Chemistry and Biochemistry, University of Wisconsin-Milwaukee, Milwaukee, WI 53211 USA; 3 Coppe Laboratories, W229N1870 Westwood Dr, Waukesha, WI 53186 USA

**Keywords:** IFNγ, Microglia, CD4^+^ and CD8^+^ T cells, Mitochondria

## Abstract

Historically, COVID-19 emerges as one of the most devastating diseases of humankind, which creates an unmanageable health crisis worldwide. Until now, this disease costs millions of lives and continues to paralyze human civilization's economy and social growth, leaving an enduring damage that will take an exceptionally long time to repair. While a majority of infected patients survive after mild to moderate reactions after two to six weeks, a growing population of patients suffers for months with severe and prolonged symptoms of fatigue, depression, and anxiety. These patients are no less than 10% of total COVID-19 infected individuals with distinctive chronic clinical symptomatology, collectively termed post-acute sequelae of COVID-19 (PASC) or more commonly long-haul COVID. Interestingly, Long-haul COVID and many debilitating viral diseases display a similar range of clinical symptoms of muscle fatigue, dizziness, depression, and chronic inflammation. In our current hypothesis-driven review article, we attempt to discuss the molecular mechanism of muscle fatigue in long-haul COVID, and other viral diseases as caused by HHV6, Powassan, Epstein–Barr virus (EBV), and HIV. We also discuss the pathological resemblance of virus-triggered muscle fatigue with myalgic encephalomyelitis/chronic fatigue syndrome (ME/CFS).

**Graphical Abstract:**

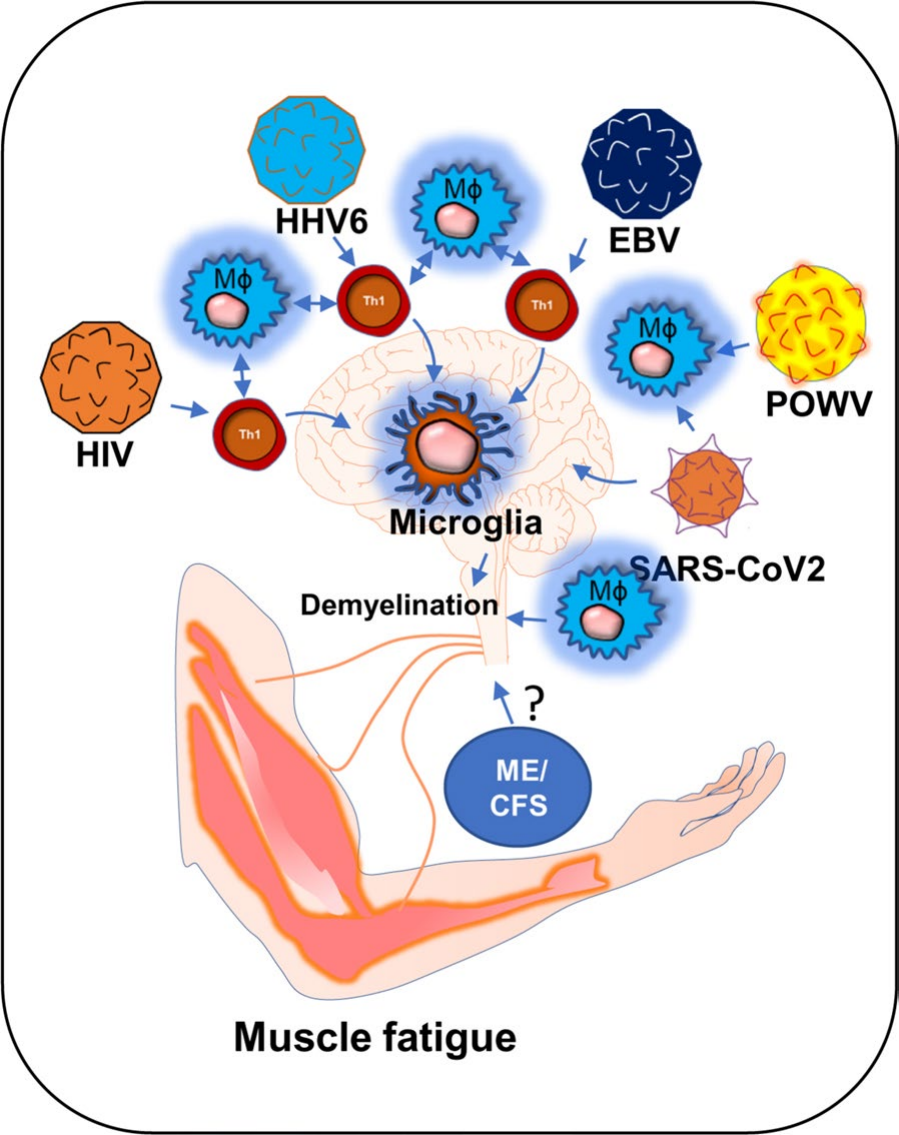

## Introduction

The role of viral infection in muscle fatigue has been debated for a long in the field of ME/CFS [1]. ME/CFS is a chronic inflammatory disease characterized by severe muscle weakness, fatigue, pain, lightheadedness, and brain fog [2]. One of the most debilitating symptoms of ME/CFS is post-exertional malaise (PEM), in which a patient suffers severe muscle fatigue and cognitive-, and orthostatic- exertions after mild exercise. This severe worsening of symptoms can cause a patient to be bedridden for a long time ranging from 24 h to several months [3, 4]. Although, the underlying molecular mechanism of severe muscle fatigue in ME/CFS is not known, a growing body of evidence suggests that intracellular inflammation and exaggerated productions of inflammatory mediators might contribute to the pathogenesis of muscle fatigue via promoting the degeneration of skeletal muscle cells and also inhibiting the differentiation of muscle progenitor cells [5, 6]. However, it is not known how the inflammation is initiated. In this context, a “ hit-and-run” mechanism of viral infection could be critical in which a transient viral infection is considered to potentiate a series of inflammatory events causing a sustained immunological disturbance [7]. A “virus reactivation theory” could be another mechanism [8], which suggests that the reactivation of viruses including EBV and HHV6 followed by a cascade of inflammatory events might contribute to the pathogenesis of ME/CFS [1]. Despite these competing hypotheses, the role of viral infection in the pathogenesis of muscle fatigue cannot be disregarded. Interestingly, a recent pandemic of COVID-19 also exhibits persistent symptoms of fatigue and weakness in approximately 10% of its survivors reiterating the potential role of virus infection in the pathogenesis of chronic fatigue syndrome [9]. Our current speculative review article discusses how HHV6, Powassan, EBV, HIV, and SARS-CoV2 viral infections adopt a common immunological mechanism that possibly leads to the debilitating muscle fatigue.

### HHV6 and chronic fatigue syndrome

**Fig. 1:**
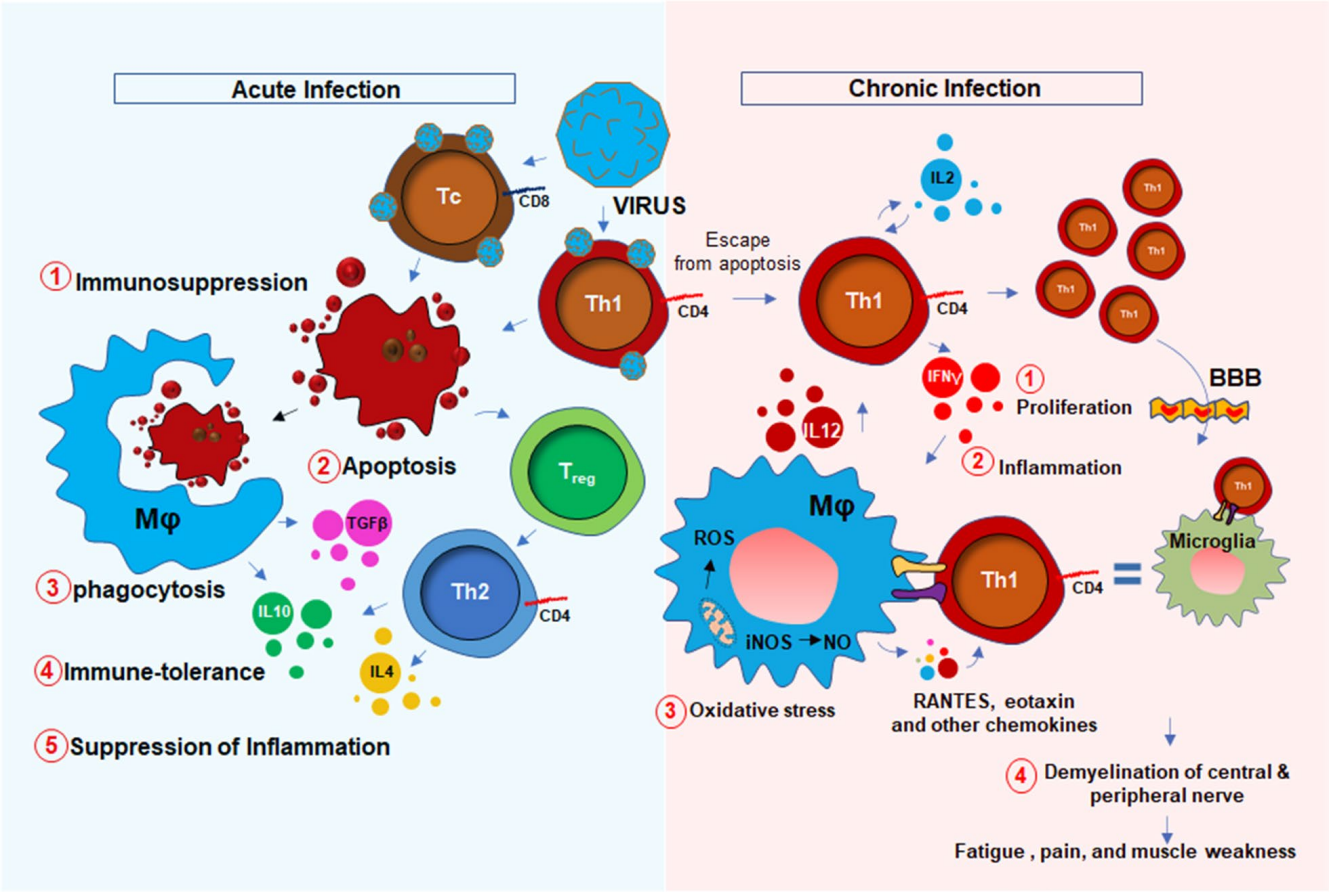
Potential mechanism of muscle fatigue is related to acute immunosuppressive and chronic inflammatory mechanisms of HHV6 viral infection. Acute infection of HHV6 (Blue shade) causes immunosuppression. During that phase, virus-infected CD4 + Th1 and CD8 + Tc cells undergo apoptosis (#1) following phagocytosis (#2) by macrophages (Mφ). During phagocytosis, Mφ release TGFβ and IL10 as a part of the immune tolerance response (#3). IL10 and IL4 are also secreted from Th2 cells during activation of FOXP3^+ve^ regulatory T cells. Together, there is an immunosuppressive response marked with reduction of inflammatory cytokines (#4). However, during chronic inflammation and viral reactivation (Red shade), a subset of persistently infected Th1 cells escape apoptosis, undergo clonal proliferation (IL2 and IL12) (#1), engage in crosstalk with Mφ, build up inflammatory milieu (#2), generate oxidatively (ROS = reactive oxygen species) and nitrosative stress (NO = nitric oxide) (#3). These inflammatory T cells also infiltrate through the blood–brain barrier (BBB), interacts with microglia causing CNS inflammation, demyelination of oligos, demyelination of nerve fibers (#4), and finally leads to the impaired nerve conduction, muscle weakness, and fatigue. FOXP3 = forkhead box P3; A master transcription factor in the development and function of regulatory T cells

The potential association between HHV6 and chronic inflammation was first introduced in 1992 by Buchwald et al. [10] when a cohort of 259 HHV6-infected patients was diagnosed with severe lymphocytic activation and cognitive impairment. Although, that study was controversial [11] to prove the link between chronic fatigue syndrome (CFS) and HHV6, in the same year, Kato et al. [12] reported a case study with a 31-year-old woman who was initially admitted with CFS, was turned out to be positive with a high titer of anti-HHV6 antigen. Later on, a PCR-based study [13] identified strong upregulations of HHV6 A and B mRNAs in 7 of 13 CFS patients with high titer of HHV6 early antigen demonstrating a strong correlation between HHV6 infection and CFS. Furthermore, a strong upregulation of IgM antibody against HHV6 early antigen (EA) [14] in 93 of 154 CFS patients (60%) [15] established another possible link between HHV6 and CFS. Although the molecular mechanism of HHV6 infection and fatigue was still unclear, HHV6 was known to induce an acute immunosuppressive response. Although both HHV6-A and-B strains infect CD4 ^+^ T helper and CD8^+^ cytotoxic T cells [16], upon infection, HHV6 selectively suppresses the expression of IL12 and inhibits Th1 polarization of CD4 ^+^ T cells [16]. In infected CD4 ^+^ T cells, HHV6 also suppresses the proliferative response by downregulating the expression of IL2 [17] and augmenting cell cycle arrest [18]. All these events induce apoptotic signals to CD4^+^ T cells (Fig. 1). In response to these apoptotic T cells, macrophages perform phagocytosis and augment an anti-inflammatory “immunotolerant” microenvironment characterized by high levels of TGF-β and IL-10 [19]. In addition, a death response to CD4 ^+^ T cells causes acute suppression of anti-viral IFN-γ production [20, 21]. Interestingly, reduced IFN-γ and increased IL-10 are historically known to suppress inflammation [22, 23]. Therefore, the role of acute HHV6 infection in inducing inflammation seems elusive. One potential mechanism could be the escape of persistently infected CD4^+^ T cells from the above-mentioned acute apoptotic pathway that potentially stimulate an inflammatory response in macrophages and glial cells. A recent report also suggests that HHV6A directly stimulates the inflammation and migration of microglial cells via activation of TREM2 and ApoE [24].Therefore, active HHV6 infection, but not an acute immunosuppressive event, may be directly responsible for responsible for the microglial activation [25], and possible demyelination [26]. Furthermore, a recent study [27] identified that patients with demyelination in CNS displayed HHV6-immunoreactive oligoclonal bands in their cerebrospinal fluid indicating a potential link between multiple sclerosis (MS)-like encephalopathy and HHV6-infection.

Nevertheless, the upregulation of 8-hydroxy-2′-deoxyguanosine (8h2dg) [28], a DNA stress marker, in the CSF of HHV6 encephalopathy patients and a significant recovery after FDA-approved ALS-drug Edarvone further confirmed the presence of encephalopathic response in HHV6 patients. That study demonstrated that 43.7% of HHV6 encephalopathy subjects had higher 8h2dg. Clinical symptoms such as muscle fatigue, sleep disturbance, problems with balance, impaired mobility, and seizures are pathological hallmarks of an encephalopathy [29, 30]. Therefore, combined with the mechanism of persistent immune activation, HHV6 infection could also trigger a CNS-specific stress response resulting in microglial inflammation [24], demyelination [31], oxidative stress [32], and neuronal damage [33], which might lead to the clinical manifestations of cognitive deficit, emotional disabilities, and muscle fatigue. HHV6 infection directly or indirectly triggers neurodegeneration. In an indirect mechanism, HHV6A promotes microglial expression of amyloid beta (Aβ) [24], the secretion of phospho-tau [24], and the induction of IL-1β. Moreover, HHV6 directly causes apoptosis of cerebellar Purkinje cells [34] suggesting its direct role in neurodegeneration. As a mechanism, the disruption of TLR4 signaling [35] and activation of TLR9 [36] followed by activation of nuclear factor κB (NF-κB) [37] might play key roles in inducing pro-inflammatory signaling events. HHV6 infection also profoundly contributes to central and peripheral demyelination. HHV6 virions directly infect oligodendroglial progenitor cells (OPCs) and cause cell cycle arrest at G1/S phase and inhibit its maturation to oligodendrocytes [38]. Other reports suggest that HHV-6A latency gene U94 directly inhibits migration and myelination of OPCs [39]. Similar to the situation in the CNS, HHV6 also induces peripheral demyelination by direct infection of the peripheral nervous system in dorsal sensory ganglia [40, 41].

Taken together, both the central and peripheral mechanisms of HHV6-induced demyelination result in the progressive loss of nerve conduction to the synaptic terminal at the neuromuscular junction resulting in the muscle weakness and fatigue (Fig. 1).

### Powassan virus encephalitis and chronic fatigue

**Fig. 2:**
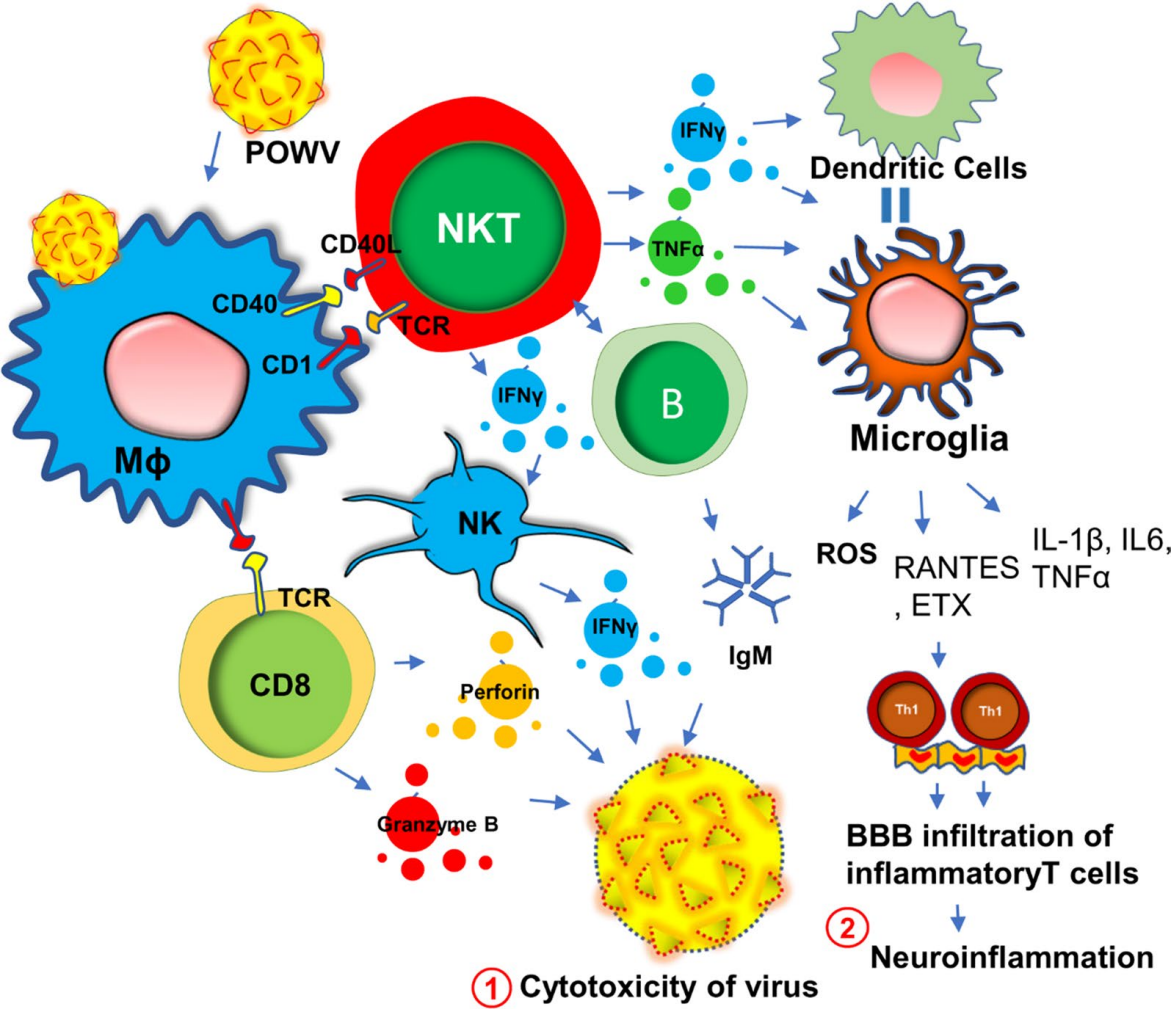
POWV infection and innate immune response for the neuroinflammatory response. Powassan virus (POWV) directly infects Mφ at early onset causing indirect activation of natural killer (NK), NKT, CD8^+^ T, and B cells. That infection triggers a protective innate immune response that results in the production of IFNγ, IgM antibodies, and cytolytic proteins including perforin and granzyme B. These factors together cause cytotoxicity of POWV particles (#1). Excessive production of IFNγ turns on the activation of microglial cells. Subsequent release of chemokines attracts inflammatory Th1 cells through the blood–brain barrier (BBB) and causes a demyelinating response in CNS (#2)

Powassan virus (POWV) encephalitis was first reported in 1958, when the titer of POWV, a neuroinvasive arbovirus, was detected from the brain autopsy of a young boy who died in Powassan, Ontario [42]. It is a tickborne flavivirus-induced [43, 44] disease that displays a wide spectrum of neuroinflammatory responses [45] in the brain and spinal cord including compromised blood–brain barrier integrity, enhanced infiltration of inflammatory T cells [46, 47], severe microglial activation [47], and demyelination [48] of oligodendrocytes resulting neuronal toxicity. While it is not known if POWV can induce a similar acute immunosuppressive mechanism as seen in HHV6, a recent study [49] demonstrated that there is a robust proliferation of reactive Th1 cells in the spleens of the POWV-infected mice. This finding suggests that, in contrast to HHV6, POWV acutely induces the inflammatory response in the early phase of infection. During the acute phase of infection, there is an activation of innate immunity (Fig. 2) for the protection against POWV infection. One such mechanism includes the activation of B cells and the subsequent expression of IgM antibodies. Indeed, elevated IgM antibodies have been identified in both CSF and sera of acute POWV-infected patients [44]. IgM antibody directly induces cytotoxicity of virus-infected cells. Another protective mechanism could include the acute activation of natural killer (NK) cells and natural killer T (NKT) cells [50] followed by the release of anti-viral cytokine IFNγ (Fig. 2). Although this mechanism is yet to be established in POWV infection, another tick-borne bacterial disease, namely Lyme disease [51] has been shown to directly activate NK cells in tick-borne encephalitis. Although, direct association of POWV with NK cells has yet to be established, infections of other flaviviruses such as West Nile virus (WNV), dengue virus (DENV), yellow fever virus (YFV), Japanese encephalitis virus (JEV), and tick-borne encephalitis virus (TBEV), have been shown to cause direct activation of NK cells [52]. However, POWV directly infects macrophages (Fig. 2) at an early stage in the tick-feeding site [53, 54], which potentially triggers the activation of NK and NKT cells to produce IFNγ causing a cytotoxic response in POWV virions (Fig. 2). The activation of cytotoxic T cells followed by the secretion of perforin and granzyme B could be another mechanism [55] for the cytotoxicity of virus-infected cells (Fig. 2). However, acute infection followed by sustained activation of innate immune response and IFNγ production could activate antigen-presenting cells as well. The activations of macrophages, dendritic cells, and microglia due to severe IFNγ production, could switch on downstream cell-based adaptive inflammatory response causing severe neuroinflammation (Fig. 2).

Similar to other tick-borne diseases such as Lyme disease, acute POWV illness presents with a diverse spectrum of clinical symptoms [56] including fever, pain, headache, and muscle weakness. Treatment paradigms are largely symptomatic and supportive thus contributing to the unpredictable course of illness over time. Interestingly, one of the most common clinical manifestations of POWV encephalitis is muscle fatigue. Encephalitis is often associated with increased demyelination [57] of peripheral nerves [58] that in turn causes impairment of ion conduction through sensory neurons [59] resulting in abnormalities in neuromuscular function [60].

Based on a recent statistical report of CDC [61], half of the people who survive severe POWV encephalitis continue to suffer from long-term muscle weakness and fatigue following their acute infection phase. Sometimes, a severe and chronic infection of POWV can cause complete paralysis in one side of the body, described clinically as hemiplegia [61]. Complete ophthalmoplegia [61] with loss of eye muscle function in both eyes is also common in POWV patients. A detailed electroencephalogram (EEG) study indicated that severe demyelination of white matter in the temporal lobe that may contribute to the loss of downstream neuronal function controlling peripheral muscle movement. Another literature reports significant infiltration of POWV in the ventral horn of the spinal cord [62] that may also contribute to the demyelinating response in the peripheral nervous system and be a potential cause of severe weakness of peripheral muscle tissue observed clinically. Taken together, these reports suggest that muscle fatigue in POWV-infected patients is possibly the result of a combination of factors including a severe demyelinating response in both the brain and spinal cord, increased expression of IFNγ, the infiltration of inflammatory T cells through the BBB, and microglial activation.

### Epstein–Barr virus (EBV) infection and muscle weakness

**Fig. 3:**
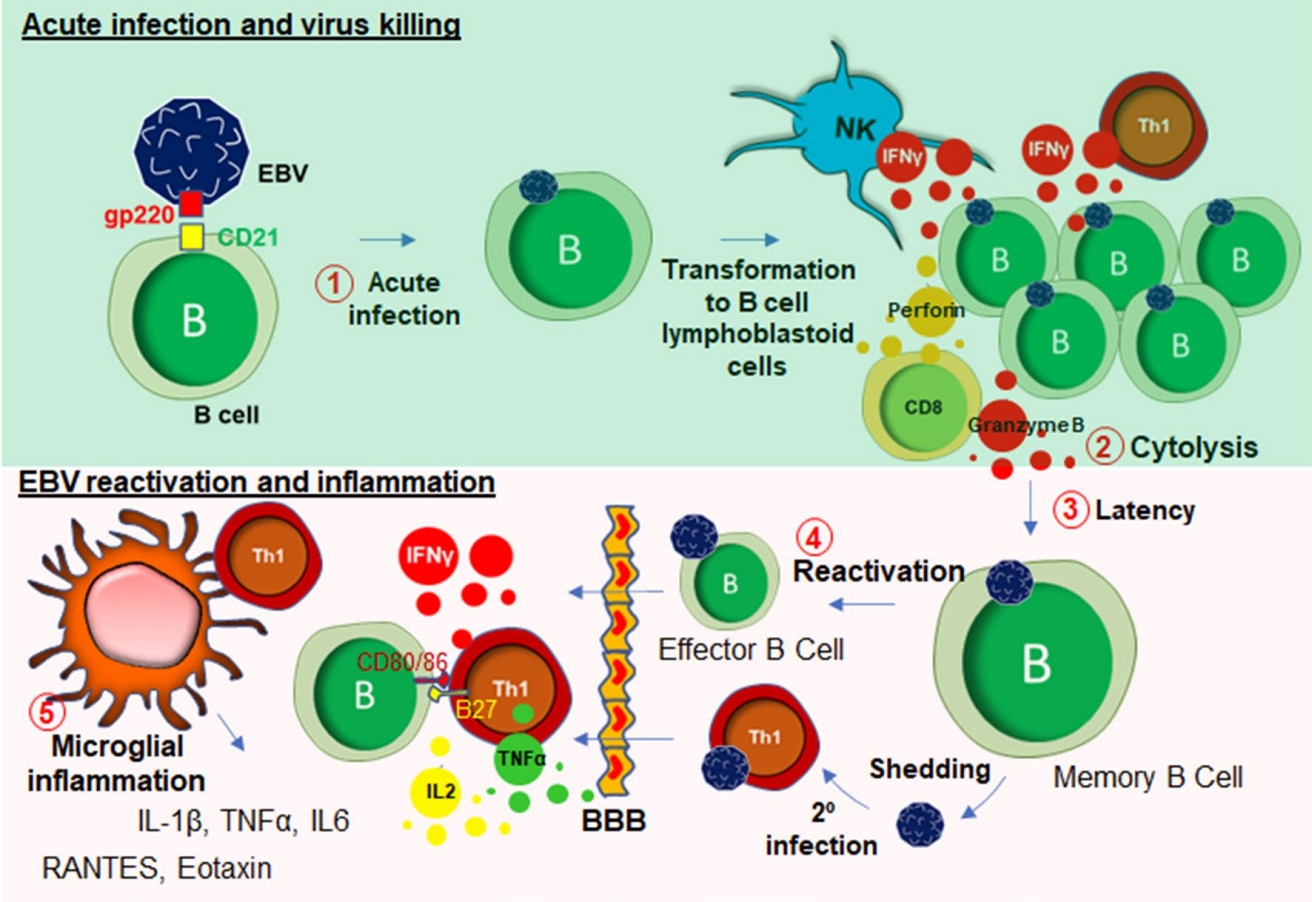
EBV infection and inflammation. EBV engages in an interaction with B lymphocyte through its gp220/350 receptors to B cell surface glycoprotein CD21. This interaction facilitates acute infection of EBV in B cells (#1), which subsequently causes transformation to B cell lymphoblastoid cells. After that, these lymphoblastoid B cells undergo cytolysis (#2) by NK cells, CD8^+^, and CD4^+^ T cells. Some B cells escape that cytolytic process and go to the latency (#3). During the late stage of life, virus reactivation (# 4) might occur followed by virus shedding, and secondary infection to Th1 cells. These reactivated and infected B and T cells possibly enter to CNS through BBB, and potentially engage in a microglial activation to induce inflammatory reactions (#5)

EBV is a DNA herpes virus that primarily spreads through oral secretions and infects resident B lymphocytes (Fig. 3) in the oropharyngeal epithelium [63]. Upon infection, EBV transforms B cells to B cell lymphoblastoid cells that eventually enter into the follicle, expands to form a germinal center (GC) [64]. The host’s protective response becomes very active at that stage, which elicits a cytotoxic response from NK cells, CD8^+^, and CD4 ^+^ T cells (Fig. 3). Infected memory B cells remain latent during this stage. However, following a secondary infection, these memory B cells rapidly convert to plasma B cells. Although B cells are the primary target of EBV infection, T cells can also be infected by EBV [65]. These lymphocytes can penetrate BBB [66] and engage with microglia (Fig. 3). In some cases, EBV directly infects microglia [67]. Upon infection, extrachromosomal episomes of EBV [68], modulate the host immune response by triggering the expression of a wide range of inflammatory cytokines such as IFN-γ, TNF-α, and IL-2 [69], NF-κB [70], and proliferation of inflammatory T lymphocytes. Another possible mechanism of CNS inflammation is molecular mimicry, by which homology between EBV nuclear antigen-1 (EBNA-1) and host’s own myelin basic protein (MBP) elicits the activation of autoreactive T cells [71]. While most EBV infections are asymptomatic, infections during adolescence and adulthood frequently cause reactivation and mononucleosis [72]. Over 50% of patients with infectious mononucleosis manifest the triad of fever, lymphadenopathy, and pharyngitis [73]. Other symptoms include splenomegaly [74], hepatomegaly [75]. Leucocytosis, atypical lymphocytosis, and elevated liver enzymes are also reported during EBV infection [76].

Recent studies demonstrate that muscle pain and fatigue can follow EBV infection and remain following the resolution of other acute symptoms. According to White et al. [77], in a cohort of 108 subjects, a subset of patients with EBV-induced glandular fever having throat and neck gland swelling was reported to display a distinct physical and mental fatigue, excessive sleep, psychomotor retardation, poor concentration, and anhedonia. The direct association of EBV infection and the pathogenesis of myalgic encephalomyelitis and chronic fatigue syndrome (ME/CFS) has been reported anecdotally for many years, and more clearly following the identification of increased EBV induced gene 2 (EBI2) expression in PBMC samples from a subgroup of ME/CFS patients [78].Moreover, upregulations of EBI2-associated early growth response genes known as EGR1, EGR2, and EGR3 in PBMCs of ME/CFS patients further reinforced the hypothesis that EBV infection could be directly linked to long-term muscle fatigue and pain experienced by the patient population [79]. In line with this idea, previous studies using animal models demonstrated that physical stress-induced immobility and restraint, may cause the upregulation of EGR1 and other immediate early genes in the CNS [80, 81]. Chronic EBV infection is often reported in patients with polymyalgia rheumatica with periodically disabling fatigue [82], and patients with primary fibromyalgia with progressive symptoms of fatigue [83]. According to a recent case study [84], EBV-infected CD8^+^ cytotoxic T cells were found to have infiltrated in the skeletal muscle tissue of 19 years old male suffering from chronic and active EBV infection suggesting a direct role of EBV infection in cytotoxicity of skeletal muscle tissue. In some patients, acute EBV infection also caused severe myocardial necrosis with marked lymphocytic infiltration [85] suggesting a direct role of EBV-infected CD8 + T cells in acute cytotoxicity [86] of cardiac tissue [87]. Although, it is not yet completely understood how EBV infection may be responsible for the development of long-term muscle fatigue, there exists clinical evidence for the development of other chronic illness [88] following acute of EBV infection including multiple sclerosis (MS) [89, 90] and, to some extent, systemic lupus erythematosus (SLE) [91, 92]. Taken together, it is now becoming evident that EBV infection and its subsequent reactivation in humans can result in the potentiation of a chronic inflammatory response in peripheral muscle tissue, and furthermore the infiltration of infected peripheral lymphocytes into the CNS.

These events eventually lead to the presentation of the cardinal clinical symptoms of ME/CFS which include fatigue, muscle weakness, dysautonomia and neurocognitive impairment. The potential relationship between chronic EBV infection and MS-like encephalopathy was further corroborated with a study by Jilek et al. [93], in which a patient with acute EBV infection was reported to display a severe myelin oligodendrocyte glycoprotein (MOG)-specific immune response accompanied with clinical signs of encephalopathy.

Collectively, muscle fatigue is a common clinical manifestation of EBV infection and reactivation and there exist multiple potential molecular pathways that may underlie clinical symptoms including the infiltration of peripheral EBV-infected CD4^+^ T cells followed by reactive microgliosis, oligodendroglial demyelination, the direct infiltration of CD8 + T cells and the subsequent cytotoxic response that might cause the weakness in in skeletal muscle tissues (Fig. 3).

### Human immunodeficiency virus (HIV) infection and muscle weakness

**Fig. 4:**
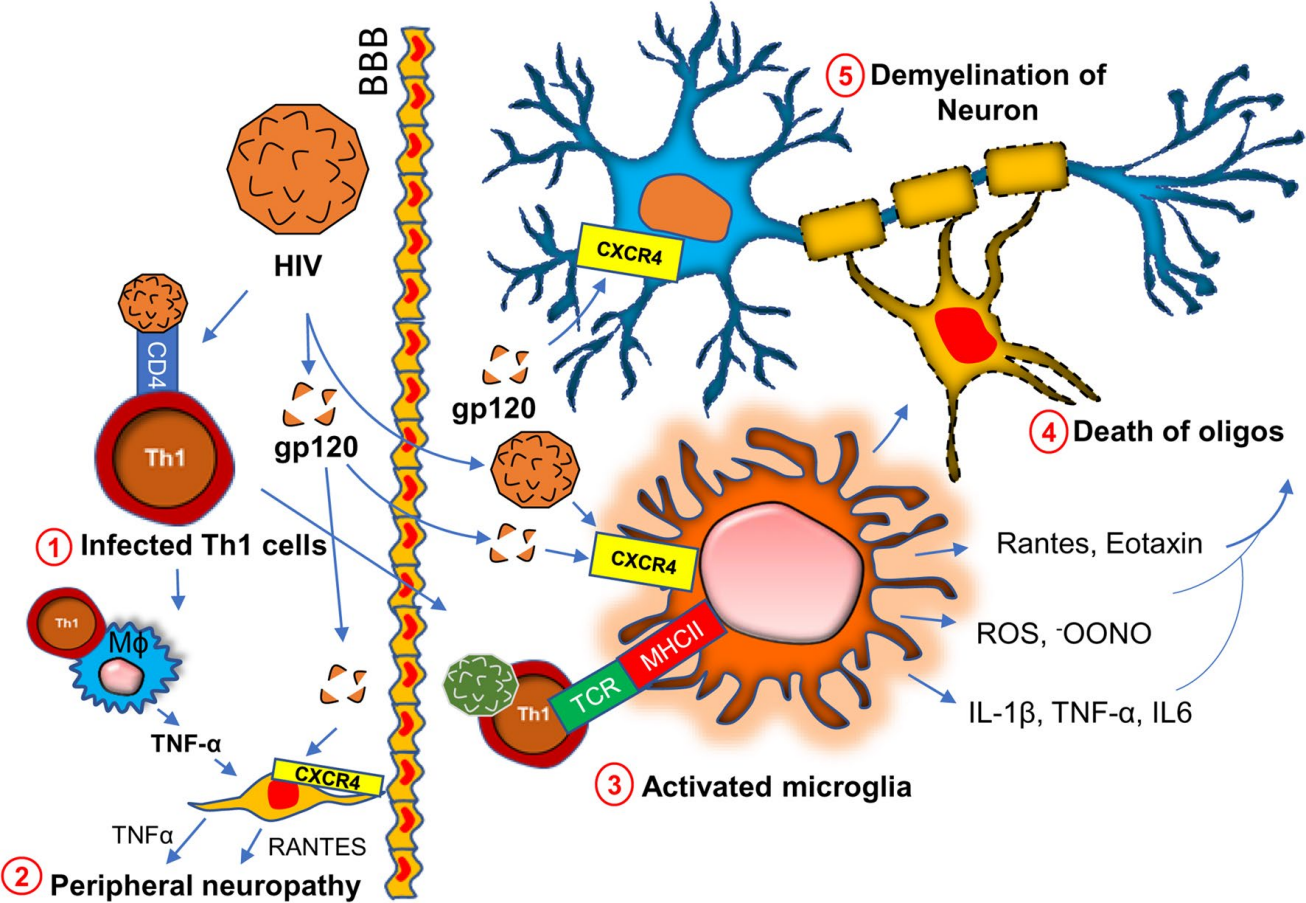
Chronic HIV infection in neuroinflammation and demyelination. HIV directly infects CD4 ^+^ T lymphocytes (#1). Infected T cells interact with macrophages causing the production of inflammatory cytokines (IL-1β, TNFα, IL6, and IL12), chemokines (CCL2. Rantes, CXCL12), reactive oxygen species (ROS), and nitric oxide. These factors together contribute to the death of Schwan cells and therefore cause peripheral demyelination (#2). HIV virions and surface protein gp120 also contribute to CNS pathology by direct interaction with microglia (#3). Subsequent production of inflammatory molecules directly causes the death of oligodendrocytes (#4) (abbreviated as “oligos”) followed by demyelination and neurodegeneration

Chronic HIV infection is often associated with severe progressive neuromuscular weakness resulting in a steady decline of muscle strength [94] and muscle mass [95], which can lead to the chronic movement impairment [96–98] and debilitating long-term disability. As a molecular mechanism, mitochondrial abnormality [99] has been often cited in muscle tissue of HIV patients. Studies have identified HIV RNA in mitochondria of mitochondria of muscle tissue collected from acute HIV-infected patients [100]. The HIV tat protein has been shown to bind and alter mitochondrial membrane potential inducing mitochondrial death [101] and is a noteworthy molecular mechanism that may underlie the clinical features of severe fatigue and a loss of muscle tissue in these patients. A specific interaction between the HIV viral protein R and the mitochondrial permeability transition pore complex (PTPC) has recently been demonstrated by Jacotot and colleagues [102]. In their work they found that PTPC-dependent permeabilization of mitochondrial membrane activates apoptosis and cytotoxicity [103] in muscle tissue. Apart from a mitochondrial impairment, a chronic inflammatory response such as activation of inflammatory T cells, gliosis, and demyelination are also critical factors [104–107] for the progression of neuromuscular weakness in HIV patients (Fig. 4). HIV virions directly infect macrophages [108] and microglia [105] and upregulate the expressions of inflammatory cytokines such as IL-1β, IL6, and TNF-α [109]; and chemokines such as CCL2, CCL5, and CXCL12 [110, 111]. Expressions of other neurotoxic factors such as NO [112, 113] and ROS [114] are also stimulated through this pathway. These factors contribute to the apoptosis of oligodendrocytes, the primary myelinating cells in CNS. Study suggests that the severity of myelin damage and white matter abnormality is often positively correlated with the microglial activation [115]. Oligodendrocytes provide critical trophic support to the neuronal cells by covering axons with myelin membranes, which is crucially important for maintaining cellular functions and electrical conduction [116]. Therefore, microglial activation [117] followed by oligodendroglial injury [118] indirectly triggers neuronal damage in HIV patients [119, 120].

In a direct mechanism, HIV surface protein gp120 has been shown to interact with neurons [121] via the CXCR4 receptor. Upon interaction, gp120 stimulates the activation of NF-κB [122] in the neuron. GP120-mediated activation of NF-κB is reported to produce ROS [123] and stimulates the formation of rod-shaped actin-cofilin conjugated proteinopathic inclusions [121] causing neurodegeneration. In the peripheral nervous system, Schwan cells also undergo apoptosis via similar mechanism. The interaction between CXCR4 of Schwan cells and gp120 of HIV causes exocytosis of lysosome and release of ATP [124]. Gp120 also triggers the release of TNFα upon binding to CXCR4 on Schwan cells [125]. TNFα potentially stimulates TNFR1-mediated apoptosis in Schwan cells and peripheral neurons causing neuropathy.

### Long-haul COVID and chronic fatigue

**Fig. 5:**
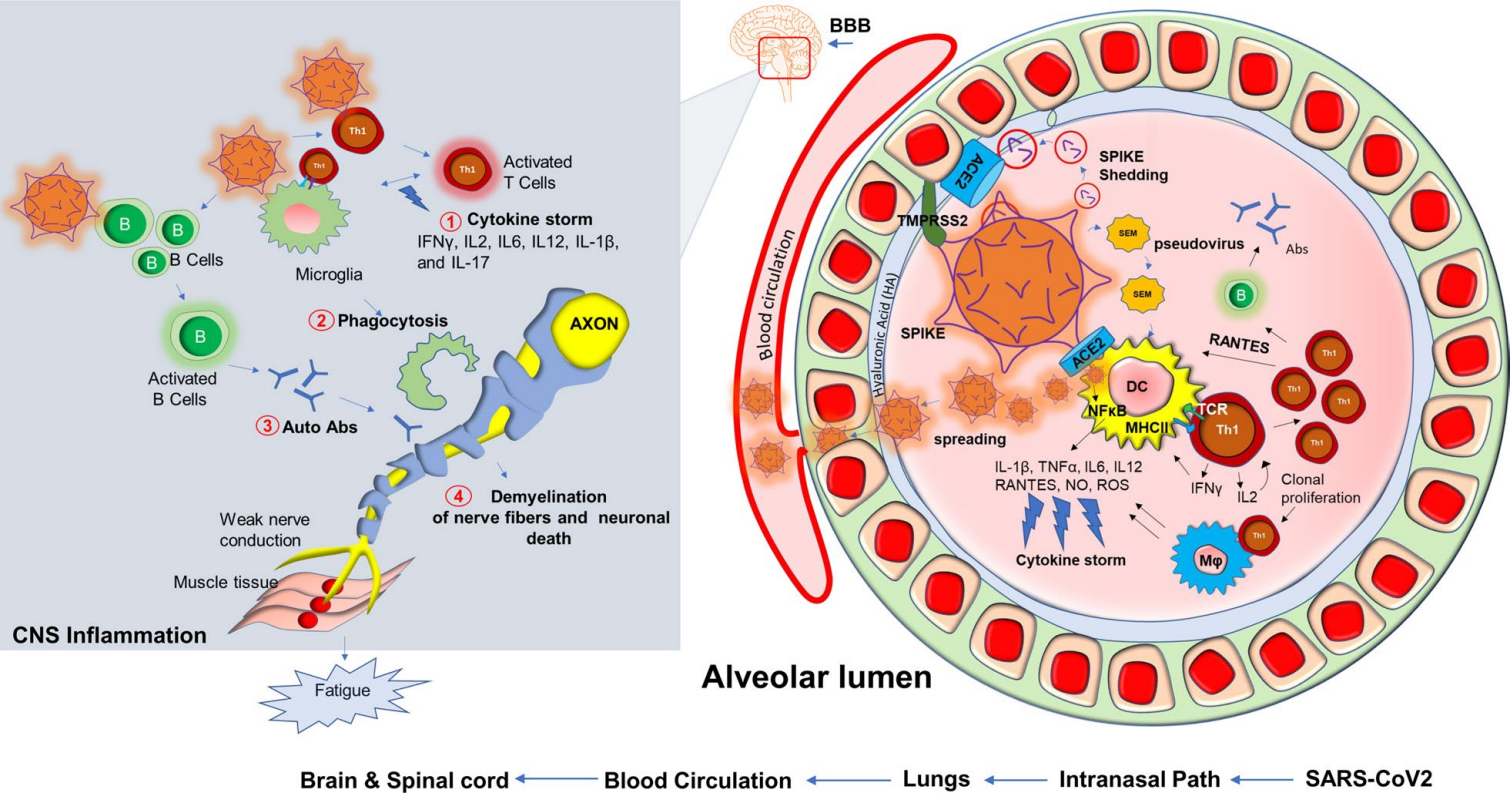
Potential inflammatory pathways in muscle fatigue of long-haul COVID patients. Upon entry of SARS-CoV2, a possible cascade of acute inflammatory pathways in the alveolar lumen was displayed. SARS-CoV2 employs its Spike protein or S-glycoprotein to bind with ACE2 receptor and membrane-bound serine protease TMPRSS2. SARS-CoV2 also interacts with hyaluronan or hyaluronic acid of the glycocalyx layer. SEM (Spike, Envelope, Membrane) pseudovirus particles or potential possible shedding of spike proteins also cause direct infection in alveolar dendritic cells followed by MHC-II presentation and activation of CD4 + Th1 cells. Subsequent production of IFNγ and virus-induced activation of NF-κB might evoke productions of inflammatory cytokines and chemokines commonly known as cytokine storm (#1). Th1 cell-mediated severe activation of Mφ and microglia might also cause non-specific phagocytosis of myelin (#2). Possible activation of B cells produces autoantibodies (#3). Eventually, active virus particles, T cells, and inflammatory mediators spread through distant organs across BBB, and cause a cell-based inflammatory response resulting demyelinating effects in the central and peripheral nervous system (#4). Impaired nerve signal causes muscular fatigue. B = B cells; T = T cells; Abs = antibodies; APCs = antigen-presenting cells

Based on our present research experience [126], dealing with the SARS-CoV2 virus in the laboratory is an exceptionally challenging and unique experience when compared to other similar RNA viruses. Potential mechanisms such as increased transmissibility [127], immune escape [128], diagnostic failure [129], and reduced effectiveness of vaccines have resulted in the development of novel variants [130] that contain rather significant mutations all of its four protein domains spike (S), envelope (E), membrane (M) and nucleocapsid (N). Mutations in these protein domains have been shown to alter an individual strain’s infectivity and transmissibility in the community. Perhaps most concerning are variants containing mutations in the S protein and the variants subsequent ability to evade approved vaccines and other treatment modalities [131]. SARS-CoV2 employs a multilayered mechanism to corrupt host cells (Fig. 5). These potential mechanisms include but are not limited to the binding with ACE-2 receptor followed by internalization in the alveolar epithelium [132–134]; infection after active engagement with transmembrane protease TMPRSS2 [135, 136]; inflammation in endothelial glycocalyx followed by disruption of hyaluronic acid [137]; “shedding” of spike protein [138] followed by insertion to the host membrane via exosomal vesicles spreading infection through spike (S), envelope (E) and membrane (M) protein enclosed pseudovirions [139]; a direct and rapid transcription of viral proteins from its positive-strand RNA; integration of gene material with host genome [140, 141] upon entry to the cells followed by exploiting host’s gene synthesis machinery [142]; taking over cellular metabolic processes of protein translation [143] and transport, and finally augmenting a “cytokine storm” [144] via synthesis of inflammatory cytokines and chemokines (Fig. 5). COVID-19 displays complex and multifaceted pathological outcomes corrupting almost every organ of the human body. As a result, COVID-19 is not only a viral disease, but its pathological significance might stretch to chronic inflammation [145–148], autoimmunity [149, 150], cancer [151], and neurodegeneration [152–154].

Although COVID-19 is significantly associated with death, 10% of total survivors display a chronic pathology that includes fever, weakness, and muscle fatigue. These symptoms are combinedly known as post-acute sequelae of COVID-19 (PASC); commonly referred to in the literature and here as long-haul COVID patients (Long haulers). “Long haulers” [155] are mostly PCR negative for COVID-19 [156], despite lingering symptoms. Although the underlying mechanism is still unknown, based on the history of viral inflammatory diseases, it is expected that dysregulation of the adaptive immune response [157, 158] could be one critical component of disease progression. Activation of CD4^+^ Th1 cells upon SARS-CoV2 infection and subsequent production of anti-viral cytokine IFN-γ might be beneficial (25) for initial virus killing; however, prolonged activation of these T cells might result in the development of a pathological inflammatory response (Fig. 5) including an elevated production of chemokines and cytokines released activated macrophages and microglia. These soluble factors recruit and engage Th1 cells on microglia followed by microglial activation causing demyelination of neuronal fibers, sensory weakness, and potentially muscle fatigue. In support of that possibility, SARS-CoV2 patients may experience a “cytokine storm” characterized by upscaled productions of inflammatory cytokines [159] such as IL2, IL12, IFN-γ, IL6, and TNFα. Several case reports also highlighted the potential demyelinating response [160–163] in SARS-CoV2 infected patients. A case study [160] revealed that a 54 year old SARS-CoV2 infected woman was admitted to the hospital after seizure. An MRI scan revealed multiple active demyelinating lesions in the brain with numerous periventricular white matter abnormalities. Hyperintense white matter abnormalities were also observed in the upper spinal cord. In another case [162], a 21-year-old post-COVID-19 patient, who met the clinical criteria for PASC and Long-haul COVID-19, was admitted following intermittent vomiting and malaise for 4 days. A subsequent brain MRI revealed the presence of bilateral posterior internal capsule lesions and longitudinally extensive transverse myelitis (LETM) in the upper spinal cord. Combining the evidence showing an exaggerated production of inflammatory cytokines, the demyelinating response in the CNS, and the role of the impaired adaptive immune response (Fig. 5) might explain the observed symptoms of chronic muscle weakness, sensory abnormalities, cognitive and autonomic dysfunction that is observed in long haulers.

Although evidences to date suggest that SARS-CoV2 can mostly affect vascular and immune cells [164], few in vitro cell culture studies also reported a direct neuroinvasive property of SARS-CoV2 in iPSC-derived neurons [165] and neural progenitor cells [166], which was further substantiated by reports suggesting a direct SARS-CoV-2 infection in cortical neurons [167].

**Fig. 6:**
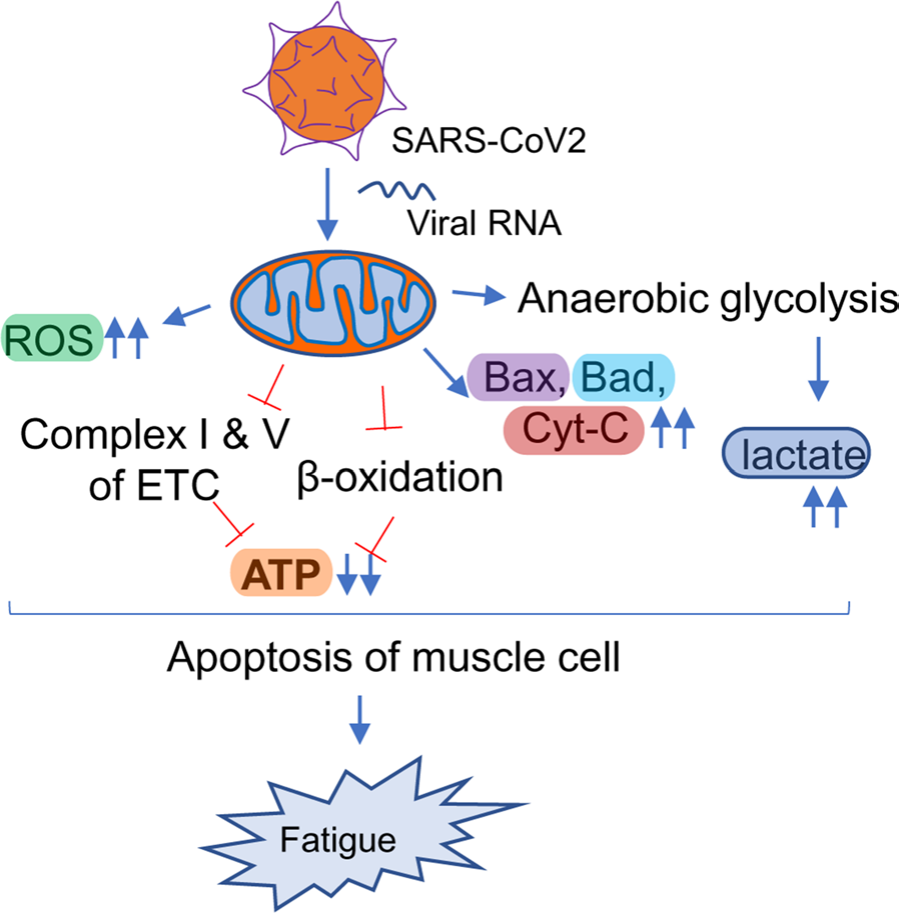
Mitochondrial impairment and its potential involvement in long-haul COVID. SARS-CoV2 directly infects mitochondria via injecting its RNA, manipulates mitochondrial gene synthesis machinery, and alters mitochondrial metabolomes. The impairment can be the release of pro-apoptotic molecules such as Bax, Bad, and cytochrome C; reversal of membrane potential; downregulation of β-oxidation and electron transport mechanism causing impaired ATP synthesis; induction of mitochondria-independent cytosolic glycolysis resulting in increased lactate synthesis. All these events trigger mitochondrial loss and eventually fatigue

Another hypothesis underlying the pathogenesis of Long haul COVID is a biochemical alteration of critical mitochondrial metabolic pathways (Fig. 6). Similar to HIV, viral RNA transcripts of SARS-CoV2 were found [168] in host mitochondria and therefore, suggests a direct role of SARS-CoV2 in in the modulation of mitochondrial function. During the acute stage of viral infection, SARS-CoV2 appears to hijack the host’s mitochondrial machinery to favor mitochondrial ATP synthesis and mitochondrial dynamics for its survival. However, chronic, or long-term viral infection is known to impair mitochondrial energy metabolism of ATP synthesis; upregulate the synthesis and release of proapoptotic molecules such as Bax, Bad, and cytochrome C; and augment ROS productions. Similarly, chronic HCV infection impairs mitochondrial energy metabolism via inhibition of Complex I and V activity [175] and decreased fatty acid β-oxidation [176]. Prolonged infection with HIV also induces mitochondrial depolarization, ROS production, and the activation of caspase 3 thus promoting accelerated mitochondrial death [177]. Chronic SARS-CoV2 infection also appears to induce the alternative energy production pathways of anaerobic glycolysis and the production of lactate [178], and thereby, potentiating muscle fatigue (Fig. 6).

### Neuroinflammation in ME/CFS

Although neuroinflammation is believed to play a critical role in the pathogenesis of ME/CFS, the molecular mechanism is still elusive. Human studies aimed to assess the contribution of inflammatory species in ME/CFS are limited, mostly due to the difficulty in obtaining cerebrospinal fluid samples and a lack of appropriately powered non-invasive imagining studies in validated cohorts [179]. Case–control studies assessing the cerebrospinal fluid collected from ME/CFS patients compared to MS comparator samples indicate a markedly disturbed pattern of CNS immune activation in ME/CFS patients with noted elevations of CCL1 (eotaxin) and an inverse relationship between interleukin 1 receptor antagonist and colony-stimulating factor 1, colony-stimulating factor 2 and interleukin 17F, without effects on interleukin 1α or interleukin 1β. Furthermore, a study [180] assessed the CSF of ME/CFS patients suggesting that CNS-specific immune dysregulation in ME/CFS patients could directly contribute to the pathogenesis. This suggests a disturbance in interleukin 1 signaling [181–183]. Interestingly, multiple studies assessing the cytokine expression patterns in peripheral blood of ME/CFS patients indicate a rather consistent signature of proinflammatory cytokine activation and an overall T helper cell type 1 pattern associated with immune activation [184–189].

Based on our published literature [190], ME/CFS serum evoked ROS and nitrite productions in cultured microglial cells. Further molecular analyses revealed that ME/CFS serum-induced production of ROS may be due to the engagement of Receptor for advanced glycation end products or RAGE. Our study also highlighted that ME/CFS patients might also demonstrate autophagy impairment that causes serum upregulations of different autophagy markers including ATG13 and alpha-synuclein. Alpha-synuclein is also known to induce microglial activation [191–193]. Both oligomeric [194] and S129P [195] alpha-synucleins induce neuroinflammatory events. Autophagy impairment directly causes mitochondrial metabolism and energy productions. Recent studies also highlight the roles of CD4 + ve and CD8 + ve T cell activation in the pathogenesis of ME/CFS [187]. Mandarano et al. have demonstrated that in ME/CFS patients, both CD4 and CD8 + T cells have reduced glycolysis and defective mitochondrial metabolism of energy.

## Conclusion

In summary, viral infection is frequently associated with muscle weakness, fatigue, and degeneration. As a molecular mechanism, alteration of adaptive immunity is widely accepted. Viruses such as EBV [196], HHV6 [197], and HIV [198] directly infect CD4 + T cells. These infected T cells proliferate and engage in a cross-talk with antigen-presenting cells (APCs) such as dendritic cells, macrophage, NK cells, and microglia (Table 1). POWV directly infects macrophages. That crosstalk stimulates the production of inflammatory cytokines, chemokine-driven recruitment of inflammatory T cells in CNS, death of oligodendroglial progenitor cells, oligodendroglial demyelination, neuronal dysfunction in the cerebellum and spinal cord resulting in diminished synaptic transmission at the neuromuscular junction. Similar to CNS, demyelinating peripheral neuropathy is frequently observed in all viral diseases. Infected CD4^+^T cells display similar inflammatory mechanisms of upregulated expressions of cytokines, macrophage activation, death of Schwann cells [199], demyelination of peripheral nerves, and muscle fatigue.

**Table 1:** Viral infections and potential mechanisms of chronic fatigue

Viral infection	Immune cells infected and activated	Potential mechanisms for fatigue
HHV6	CD4^+ve^ (infection and apoptosis) and CD8^+ve^ T cells, NK Cells, microglia (activation)	Immunosuppression [18], autoimmune reaction (IgM abs), microglial activation and production of cytokines, Amyloid-beta [24], OPC immuration, demyelination [169]
POWV	CD4^+ve^ Th1 cells (proliferation), B Cell (activation), macrophage (infection and activation)	Proliferation of Th1 cells, IgM production, microglia-induced inflammation, demyelination in peripheral nerves
EBV	B cells (infection), CD8^+ve^ T cell activation, microglia (activation)	EBV-specific CD8 + ve T cell-induced Muscle cell apoptosis [85, 87] Reactive gliosis [170], and demyelination [89]
HIV	CD4^+ve^ T cells (infection) [171], macrophages and microglia (infection)	Mitochondrial permeabilization and depolarization in muscle cells, activation of inflammatory T cells, microgliosis, and demyelination
SARS-CoV2	Lung endothelial cells (infection), kidney cells (infection), CD4^+ve^ T (infection) [172], CD8^+ve^ T cells (activation and exhaustion)	Cytokine storm (IL-1b, TNF-1, IL6 etc.)[173], Glial activation [174], and T cells exhaustion. Mitochondrial impairment, Direct Infection and toxicity to neurons

In another hypothesis, virus-infected CD8^+^ cytotoxic T cells directly infiltrate muscle tissue causing muscular degeneration, which is frequently observed in EBV and POWV infection. However, it is not known if SARS-CoV2 directly infects CD4^+^ or CD8^+^ T cells. However, SARS-CoV2 directly infects APCs such as macrophage, dendritic cells, and microglia causing a cell-based activation of CD4^+^ and Cd8^+^ T cells. Upon activation, these inflammatory T cells potentially infiltrate into the CNS and augment a series of demyelinating responses including microglial activation, death of OPCs, oligodendroglial demyelination, alteration of synaptic transmission that eventually led to muscle weakness and fatigue. In addition to that, we also discussed a biochemical mechanism of mitochondrial impairment and a chronic deficit of energy metabolism in the pathogenesis of post-acute sequelae of COVID-19. Taken together, our review article hypothesizes a mechanistic insight of chronic muscle fatigue due to long-term viral infection.

## Data Availability

There is no electronic datasheet associated with this paper. No data in electronic repository.

## Abbreviations

ME/CFS: Myalgic encephalomyelitis/chronic fatigue syndrome
PASC: Post-acute sequelae of COVID-19
EBV: Epstein–Barr virus
HHV6: Human herpesvirus 6
HIV: Human immunodeficiency virus
Mφ: Macrophage
COVID-19: Coronavirus disease 2019
NO: Nitric oxide
ROS: Reactive oxygen species
