# Correction: Potential molecular mechanisms of chronic fatigue in long haul COVID and other viral diseases

**DOI:** 10.1186/s13027-023-00505-y

**Published:** 2023-04-24

**Authors:** Carl Gunnar Gottschalk, Daniel Peterson, Jan Armstrong, Konstance Knox, Avik Roy

**Affiliations:** 1 Simmaron Research INC, 948 Incline Way, Incline Village, NV 89451 USA; 2 Research and Development Laboratory, Department of Chemistry and Biochemistry, University of Wisconsin-Milwaukee, Milwaukee, WI 53211 USA; 3 Coppe Laboratories, W229N1870 Westwood Dr, Waukesha, WI 53186 USA

**Correction to**: *Infectious Agents and Cancer (2023) 18:7*.

10.1186/s13027-023-00485-z.

Following publication of the original article [1], the authors identified an error in the affiliations assignment: affiliation 3 (Coppe Laboratories, W229N1870 Westwood Dr, Waukesha, WI 53,186, USA) was incorrectly assigned to all authors, instead of only to fourth author, Konstance Knox.

This error is corrected in the author list of this Correction article and the original article [1] has been updated.
